# Supplementary material for: Understanding the Wellbeing Needs of First Nations Children in Out-of-Home Care in Australia: A Comprehensive Literature Review
Source: Int J Environ Res Public Health. 2024 Sep 13;21(9):1208. doi: 10.3390/ijerph21091208 (PMC11431735; doi:10.3390/ijerph21091208)
Supplement: Supplementary file 1 [file ijerph-21-01208-s001.zip › ijerph-3185833-supplementary.pdf]

# Supplementary Material

**Table S1.** Peer-reviewed literature search strategy.

| Database | OOHC Terms | Wellbeing Terms | Population Terms |
|---|---|---|---|
| **PsycINFO**<br>**Scopus**<br>**MEDLINE**<br>**Informit**<br>**Embase** | **Title/Abstract search:** "out of home care" OR "out-of-home care" OR OOHC OR "out of home placement" OR "out-of-home placement" OR "residential care" OR "state care" OR "public care" OR "kinship care" OR "in care" OR "foster care" OR "foster family care" OR "foster home care" OR "foster child*" OR "guardian*" | **Title/Abstract search:** wellbeing OR well-being OR SEWB OR "quality of life" OR HR-QOL OR HRQOL OR QOL OR wellness OR "life quality" OR "health related quality of life" OR "health-related quality of life" OR "cultur*" | **Title/Abstract search:** Aborigin* OR Indigenous OR "Torres Strait" OR "First Nation*" OR "First Australia*" |
| **CINAHL**<br><br>**Expanders—**Apply equivalent subjects<br>**Narrow by SubjectGeographic:—**australia & new zealand **<br>**Search modes—**Boolean/Phrase | **Title/Abstract search:** "out of home care" OR "out-of-home care" OR OOHC OR "out of home placement" OR "out-of-home placement" OR "residential care" OR "state care" OR "public care" OR "kinship care" OR "in care" OR "foster care" OR "foster family care" OR "foster home care" OR "foster child*" OR "guardian*" | **Title/Abstract search:** wellbeing OR well-being OR SEWB OR "quality of life" OR HR-QOL OR HRQOL OR QOL OR wellness OR "life quality" OR "health related quality of life" OR "health-related quality of life" OR "cultur*" | **Title/Abstract search:** Aborigin* OR Indigenous OR "Torres Strait" OR "First Nation*" OR "First Australia*" |

Note: the truncation symbol * is used to inform the relevant database to find all variants of a given word following the symbol. For example, the term cultur* would also include searches for culture, cultures, cultured, cultural, and culturally. ** Narrow by SubjectGeographic on CINAHL is limited to include both Australia and New Zealand. No option to just include Australia.

**Table S2.** Included grey literature search sites and strategy.

| Source and Website | Search Terms | Additional Search Strategies |
|---|---|---|
| **Australian Indigenous HealthInfoNet (https://healthinfonet.ecu.edu.au/)** | Searched Out of home care AND wellbeing, with the "Child protection and care," topic filter used. Screened all 58 results. | Reviewed reference lists for relevant articles and/or reports |
| **Secretariat of National Aboriginal and Islander Child Care (SNAICC) https://www.snaicc.org.au/** | Searched "Out of home care." Screened first 5 pages. | Reviewed reference lists for relevant articles and/or reports |
| **Google Scholar** | ("out of home care" OR "foster care") AND (wellbeing OR culture) AND ("Aboriginal and Torres Strait Islander" OR Indigenous OR "First Nation"). Reviewed first 5 pages (50 results) | Cookies and cache cleared prior to searching under incognito/private browser mode |

**Table S3.** Exclusion criteria hierarchy for article screening

| Order | Exclusion Label | Exclusion Reason |
| --- | --- | --- |
| 1 | Not English | Publication is not written in English. |
| 2 | Wrong country | Publication is not conducted and/or does not include Australian participants and findings. |
| 3 | Wrong publication type | Publication **is not a** qualitative article or report (or a mixed methods study). Wrong publication types include a thesis, book or book chapter, media documents, conference presentations or abstracts, and book reviews. |
| 4 | Wrong population | The publication **does not** include First Nations Australian children in OOHC, or First Nations adults with lived experience in OOHC, or OOHC carers, caseworkers, organizational stakeholders, or First Nations Australian community members with lived and/or professional experience with OOHC.<br><br>If it is any of the above members, they could be excluded if the participant population is focusing on a specific disease or condition. |
| 5 | Wrong study type | Publication is a trial, study protocol, intervention or health service delivery study, or a quantitative study. |
| 6 | Nil wellbeing focus | Publication includes **no** primary qualitative data/findings related to the wellbeing needs of First Nations children in OOHC. |

**Table S4.** Peer-reviewed literature article characteristics.

| Reference | Aim * | Location | Method | Participant Population | Participant Perspective | Placement Type |
|---|---|---|---|---|---|---|
| Higgins et al. (2006) [43] | BROAD | Queensland and Western Australia | Focus groups | Population number: 27 carers, 16 children/youth<br>First Nations population: 9 carers, 16 children/youth<br>Gender: N/A<br>Age: N/A carers, 7–16 years old children/youth | Children/youth and carers | Out-of-home care (general), kinship care |
| Kiraly et al. (2014) [36] | BROAD | Victoria | Focus groups | Population number: 57<br>First Nations population: 15<br>Gender: 55 female, 2 male<br>Age: 23 ≤ 50 years old, 22 between 51–60 years old, 12 > 60 years old | Carers | Kinship care |
| Mendes et al. (2021) [49] | BROAD | National | Individual interviews, focus groups | Population number: 53 (32 from NGOs, 12 from ACCOs)<br>First Nations population: N/A<br>Gender: N/A<br>Age: N/A | Staff | Transitioning out of OOHC |
| Mendes et al. (2022) [48] | BROAD | National | Individual interviews, focus groups | Population number: 53 (32 from NGOs, 12 from ACCOs)<br>First Nations population: N/A<br>Gender: N/A<br>Age: N/A | Staff | Transitioning out of OOHC |
| Moss (2008) [51] | BROAD | Regional Queensland | In-depth narrative art interviews (children), staff interviews | Population number: 20 children/youth, 11 staff<br>First Nations population: 16 children/youth, 7 staff<br>Gender: 14 female, 6 male children/youth, N/A staff<br>Age: Children/youth 4-18 years old, staff N/A | Children/youth and staff | 65% of participants in OOHC, 35% never been in care |
| Raman et al. (2017) [64] | BROAD | Sydney, NSW | Individual interview, focus groups | N/A | Staff | Kinship care, foster care |
| Spence (2004) [52] | BROAD | New South Wales | Interviews (unspecified) | Population number: 9 children/youth, 9 staff, 11 kinship carers<br>First Nations population: N/A, mentions both Indigenous and non-Indigenous participated<br>Gender: N/A<br>Age: Children 5–12 years old, N/A staff/carers | Children/youth, carers, staff | Kinship care |

| | | | | | | |
|---|---|---|---|---|---|---|
| Turnbull-Roberts et al. (2021) [33] | BROAD | National | Review of Aboriginal community-controlled organisation's submissions to the 2018 Australian Senate Parliamentary Inquiry into Adoption Reform | 7 submissions from ACCOs were reviewed. Age and gender N/A | Staff | Out-of-home care (general) |
| McMahon et al. (2007) [38] | YES | Mackay, QLD | Focus groups | Population number: 20<br>First Nations population: 20<br>Gender: N/A<br>Age: N/A | Carers and staff | Foster care |
| Krakouer (2023) [58] | BROAD | Victoria | Semi-structured interview | Population number: 10<br>First Nations population: 10<br>Gender: N/A<br>Age: 15–25 years old | Children/youth (retrospective accounts of experiences in OOHC) | Out-of-home care (general) |
| Black et al. (2023) [55] | YES | Melbourne, Victoria | Semi-structured interview (flexible approach- option to provide written responses instead) | Population number: 4<br>First Nations population: 3<br>Gender: 3 female, 1 N/A<br>Age: 3 were 'early to mid 20s,' 1 N/A | Young adult retrospective, and staff | Out-of-home care (general) |
| Jau et al. (2022) [57] | BROAD | Victoria and Western Australia | Individual interviews and focus groups | Population number: 34 care leavers, 24 staff<br>First Nations population: 10 care leavers (staff N/A)<br>Gender: 22 female, 12 male (care leavers)<br>Age: 18–25 years old | Youth transitioning out of care, and staff | Transitioning out of OOHC |
| Newton et al. (2023) [60] | BROAD | NSW | Open-ended qualitative survey questions, interviews, and the Kvebaek Family Sculpture Technique | Population number: 39 parents, 96 children survey responses, >200 caseworker responses<br>First Nations population: 39 parents, 96 children, N/A caseworkers<br>Gender: 32 mothers, 7 fathers<br>Age: Children ≤ 17 years old | Parents of children who had been restored from OOHC, children/youth, and caseworkers | Out-of-home care (general) |
| Creamer et al. (2022) [56] | BROAD | Central QLD | Interviews (strengths-based yarning approach) | Population number: 28 (10 Elders, 10 staff, 8 young adults)<br>First Nations population: 28<br>Gender: Elders (6 female, 4 male), staff (7 female, 3 male), young adults (5 female, 3 male)<br>Age: Young adults were between 19-26 years old | Elders, staff, young adults who exited OOHC (retrospective perspective) | Out-of-home care (general) |

| | | | | | | |
|---|---|---|---|---|---|---|
| Roche et al. (2023) [61] | BROAD | Northern Territory | Interviews and document analysis | Population number: 28 (8 staff/carers, 20 children/youth)<br>First Nations population: 18 (1 carer/staff, 17 children/youth)<br>Gender: Carers/staff (N/A), children/youth (7 female, 13 male)<br>Age: Children/youth were 9–18 years old | Carers, staff, and children/youth | Out-of-home care (general) |
| Kor et al. (2023) [65] | BROAD | New South Wales | Focus groups | Population number: 30<br>First Nations population: N/A<br>Gender: 28 female, 2 male<br>Age: N/A | Staff (caseworkers, casework managers, and practice improvement specialists) | Out-of-home care (general) |
| Clarke et al. (2023) [62] | BROAD | Queensland | Semi-structured interviews | Population number: 20<br>First Nations population: 6<br>Gender: 20 female, 0 male<br>Age: N/A | Kinship carers | Kinship care |
| Hassall et al. (2023) [67] | BROAD | Unspecified (‘one of two major Australian cities’) | Individual Five-Minute Speech Sample (FMSS) | Population number: 66<br>First Nations population: 7<br>Gender: N/A<br>Age: 29–84 years old (M = 51.77 years) | Caregivers | Foster and kinship care |
| Wright & Collings (2023) [63] | BROAD | New South Wales | Photovoice | Population number: 11<br>First Nations population: 4<br>Gender: 9 female, 2 male<br>Age: 16–25 | Care-experienced youth, retrospectives accounts | Out-of-home care (general) |

* Wellbeing main aim of study = YES. Component broader research question = BROAD, ** Article contains same sample population as another included article.

**Table S5.** Grey literature article characteristics.

| Authors (Year) | Aim * | Location | Research Method | Participant Details | Participant Perspective | Placement Type |
|---|---|---|---|---|---|---|
| Higgins et al. (2005) [53] | BROAD | Queensland and Western Australia | Focus groups | Population number: 27 carers, 16 children<br>First Nations population: 9 carers, 16 children<br>Gender: N/A<br>Age: N/A carers, 7–16 years old children | Children/youth and carers | Foster care, kinship care, residential care |
| Kiraly & Humphreys (2011) [44] | BROAD | Victoria | Individual interviews, focus groups | Population number: 13<br>First Nations population: 11 plus 2 with Aboriginal family<br>Gender: MF (number N/A)<br>Age: N/A | Carers and staff | Kinship care |
| Terri Libesman (2011) [46] | BROAD | National | Focus groups | Population number: N/A<br>First Nations population: N/A, 8 focus groups conducted with Aboriginal and Torres Strait Islander child protection agencies<br>Gender: N/A<br>Age: N/A | Staff | Out-of-home care (general) |
| Megan Davis (2019) [37] | BROAD | New South Wales | ACCO consultation | 23 submissions reviewed from key stakeholders including peak Aboriginal advocacy bodies, academics, OOHC providers, etc. | Staff | Out-of-home care (general) |
| Lewis et al. (2019) [45] | BROAD | National | Interviews (unspecified) | Demographic variables unspecified<br>Mentions Aboriginal and Torres Strait Islander young people provided input in this report | Children/youth, staff, and community | Out-of-home care (general) |
| Moore et al. (2007) [50] | BROAD | Australian Capital Territory | Youth forums | Population number: 52 children and youth<br>First Nations population: 52 Aboriginal and/or Torres Strait Islander<br>Gender: 21 male, 31 female<br>Age: 7 to 17 years old | Children/youth | Out-of-home care (general) |
| Hermeston et al. (2016) [42] | BROAD | National | Submissions and interviews (unspecified) | Unspecified demographic variables: Input and submissions from six Aboriginal and Torres Strait Islander community-controlled organizations | Staff | Out-of-home care (general) |
| Commissioner for Children and Young People WA (2016) [34] | BROAD | Western Australia | Individual interviews, phone interviews, online survey, and focus groups | Population number: 96 children and youth<br>First Nations population: 40 Aboriginal and/or Torres Strait Islander<br>Gender: Of Indigenous participants- 22 female, 18 male<br>Age: 8 to 24 years old | Children/youth, retrospective adult | Out-of-home care (general) |

| | | | | | | |
|---|---|---|---|---|---|---|
| Advocate for Children and Young People (2021) [39] | BROAD | New South Wales | Individual interviews, focus groups | Population number: 99 children and youth<br>First Nations population: 30 Aboriginal and/or Torres Strait Islander<br>Gender: 36 male, 55 female, 2 another gender or non-binary<br>Age: 6 to 24 years old | Children/youth, retrospective adult | Out-of-home care (general) |
| AbSec (2020) [35] | BROAD | New South Wales | Interviews (unspecified) | Population number: More 50 Aboriginal young people, families, Indigenous and non-Indigenous carers, practitioners, and community members were interviewed. | Children, carers, staff, community | Out-of-home care (general) |
| Commission for Children and Young People (2016) [41] | BROAD | Victoria | Interviews (unspecified) | Population number: 980 children<br>First Nations population: 97.5% Aboriginal, >2% Torres Strait Islander<br>Gender: 51.1% female, 48.9% male<br>Age: 78.4% under 12 years old | Children | Out-of-home care (general) |
| Commission for Children and Young People (2019) [32] | BROAD | Victoria | Interviews (unspecified) | Population number: 204 children and youth<br>First Nations population: 40% Aboriginal<br>Gender: 48% female, 52% male<br>Age: 86% under 18 years old | Children | Residential care, foster care, kinship care |
| McDowall et al. (2018) [47] | BROAD | National | Survey | Population number: 1275 children and youth<br>First Nations population: 436 Aboriginal and/or Torres Strait Islander<br>Gender: 56.9% female, 43.1% male<br>Age: 60.8% between 10–14 years old, 37.3% between 15–18 years old | Children | Foster care, kinship care, residential care, permanent placement |
| Bamblett et al. (2012) [40] | BROAD | Victoria | Individual interviews, focus group | Population number: 24 staff<br>Indigenous population: 15 Aboriginal<br>Gender: N/A<br>Age: N/A | Staff | Out-of-home care (general) |
| Liddle et al. (2022) [59] | BROAD | National | Community and stakeholder consultation | * Does not specify participant demographics/number | Aboriginal and Torres Strait Islander community and sector leaders, staff | Out-of-home care (general) |
| Liddle et al. (2023) [54] | BROAD | National | Community and stakeholder consultation | * Does not specify participant demographics/number | Aboriginal and Torres Strait Islander community and sector leaders, staff | Out-of-home care (general) |

* Wellbeing main aim of study = YES. Component broader research question = BROAD, ** Article contains same sample population as another included article.
